# Ovarian Tumor Treated by Incision and Drainage

**Published:** 1881-07

**Authors:** 


### OVARIAN TUMOR TREATED BY INCISION AND DRAINAGE.

In the *New York Medical Journal* for June, 1881, Dr. T. Gaillard Thomas relates the case of a lady on whom two attempts had been made to extirpate a large ovarian tumor, which attempts were abandoned on account of the extensive adhesions encountered and the profuse hæmorrhage that took place. The patient's health became very much depreciated, and on several occasions she passed into a state of collapse, and was thought by her physician to be dying. She now entered Dr. Thomas's private hospital, where, after vain endeavors to improve her condition by great care and thorough drainage ( a drainage tube having been inserted by the surgeon who first attempted the removal of the tumor, and the opening still persisting), Dr. Thomas cut directly down upon the tumor, and without opening the peritonæum, tried to enucleate it. There was so great hæmorrhage, however, and the sac was so universally attached, that he gave it up, and cut directly into the mass, when a large amount of colloid fluid escaped. Carrying his hand into it, he found a large number of sacs, each of about the size of a cocoanut, filled with fluid, which he broke up. One existed almost outside the large tumor, and it was into that that the India-rubber tube had been inserted by the patient, and pus withdrawn. He opened this thoroughly, exercising care that none of its contents should enter the peritonœum. Two glass drainage tubes were then inserted, one above and one below, in Douglas's cul-de-sac, through which carbolized water could be injected. The patient was placed upon the most nutritious diet, and injections of carbolized water were employed. The tumor diminished in size until it was not larger than the head of a child at birth, and one month after the operation she left the hospital, with instructions to keep up the injections of carbolized water. A month later she appeared to be perfectly well, a cyst the size of a goose's egg still remaining, which she drained with perfect ease.

